# Mutations in *ompK36* differentially impact *in vitro* synergy of meropenem/vaborbactam and ceftazidime/avibactam in combination with other antibiotics against KPC-producing *Klebsiella pneumoniae*

**DOI:** 10.1093/jacamr/dlad113

**Published:** 2023-10-26

**Authors:** Tara M Rogers, Ellen G Kline, Marissa P Griffith, Chelsea E Jones, Abigail M Rubio, Kevin M Squires, Ryan K Shields

**Affiliations:** School of Pharmacy, University of Pittsburgh, Pittsburgh, PA, USA; Department of Medicine, University of Pittsburgh, 3601 Fifth Avenue, Falk Medical Building, Suite 5B, Pittsburgh, PA, USA; Department of Medicine, University of Pittsburgh, 3601 Fifth Avenue, Falk Medical Building, Suite 5B, Pittsburgh, PA, USA; Department of Medicine, University of Pittsburgh, 3601 Fifth Avenue, Falk Medical Building, Suite 5B, Pittsburgh, PA, USA; Department of Medicine, University of Pittsburgh, 3601 Fifth Avenue, Falk Medical Building, Suite 5B, Pittsburgh, PA, USA; Department of Medicine, University of Pittsburgh, 3601 Fifth Avenue, Falk Medical Building, Suite 5B, Pittsburgh, PA, USA; Department of Medicine, University of Pittsburgh, 3601 Fifth Avenue, Falk Medical Building, Suite 5B, Pittsburgh, PA, USA; Department of Medicine, University of Pittsburgh, 3601 Fifth Avenue, Falk Medical Building, Suite 5B, Pittsburgh, PA, USA; Center for Innovative Antimicrobial Therapy, University of Pittsburgh, Pittsburgh, PA, USA; Antibiotic Management Program, University of Pittsburgh Medical Center, Pittsburgh, PA, USA

## Abstract

**Objectives:**

Ceftazidime/avibactam and meropenem/vaborbactam are preferred agents for *Klebsiella pneumoniae* carbapenemase (KPC)-producing *K. pneumoniae* (KPC-*Kp*) infections and are often used in combination with other agents. We aimed to characterize the synergy of combinations against KPC-*Kp* with varying *ompK36* genotypes.

**Methods:**

KPC-*Kp* that harboured *ompK36* WT, IS*5* or glycine-aspartic acid duplication (GD) genotypes were selected. MICs were determined in triplicate. Synergy was assessed by time-kill assays for ceftazidime/avibactam and meropenem/vaborbactam in combination with colistin, gentamicin, tigecycline, meropenem or fosfomycin against 1 × 10^8^ cfu/mL KPC-*Kp*.

**Results:**

KPC-*Kp* harboured *ompK36* WT (*n* = 5), IS*5* (*n* = 5) or GD (*n* = 5); 11 were KPC-2 and 4 were KPC-3. All were susceptible to ceftazidime/avibactam and meropenem/vaborbactam. In time-kill analysis, ceftazidime/avibactam and meropenem/vaborbactam 1 × MIC exhibited mean 24 h log-kills of −2.01 and −0.84, respectively. Ceftazidime/avibactam was synergistic in combination with colistin independent of *ompK36* genotype. Ceftazidime/avibactam combinations impacted by porin mutations (compared to WT) were meropenem (−5.18 versus −6.62 mean log-kill, *P* < 0.001) and fosfomycin (−3.98 versus −6.58, *P* = 0.058). Mean log-kills with meropenem/vaborbactam were greatest in combination with gentamicin (−5.36). In the presence of porin mutations, meropenem/vaborbactam killing activity was potentiated by the addition of colistin (−6.65 versus −0.70, *P* = 0.03) and fosfomycin (−3.12 versus 1.54, *P* = 0.003).

**Conclusions:**

Our results shed new light on the synergy of ceftazidime/avibactam and meropenem/vaborbactam combinations against KPC-*Kp* with or without porin mutations. Killing activity of ceftazidime/avibactam with other cell wall active agents was decreased against isolates with porin mutations. On the other hand, some meropenem/vaborbactam combinations demonstrated enhanced killing in the presence of porin mutations.

## Introduction

The development of the novel β-lactam/β-lactamase inhibitor (BL/BLI) agents ceftazidime/avibactam and meropenem/vaborbactam has changed the therapeutic landscape against infections caused by *Klebsiella pneumoniae* carbapenemase (KPC)-producing *K. pneumoniae* (KPC-*Kp*) over the last decade.^1^ Prior to the availability of BL/BLI agents, expert guidelines recommended polymyxin-based combinations as salvage therapy against carbapenem-resistant bacteria.^1,2^ Now, both the IDSA and ESCMID recommend either ceftazidime/avibactam or meropenem/vaborbactam as monotherapy for the treatment of infections caused by carbapenem-resistant Enterobacterales (including KPC-*Kp*), without preference for either agent.^3–5^ Other recently approved agents like imipenem/relebactam and cefiderocol demonstrate excellent *in vitro* activity against KPC-*Kp*; however, real-world clinical data to support guideline recommendations are lacking.

Despite advances in treatment of KPC-*Kp* infections, reports of resistance and treatment failure, particularly with ceftazidime/avibactam therapy, continue to emerge.^6–8^ Reduced susceptibility to ceftazidime-avibactam is often mediated by mutations in *bla_KPC_*. *In vitro* data suggest that ceftazidime/avibactam is particularly vulnerable against KPC-3-producing *Kp*, which exhibit 30 times greater hydrolytic activity against ceftazidime compared with KPC-2.^9^ This mechanism has been implicated in treatment failures and selection of ceftazidime/avibactam resistance.^10–13^ Vaborbactam, on the other hand, is a cyclic boronic acid BLI designed specifically to inhibit KPC enzymes, and it demonstrates potent inhibitory activity against KPC variants resistant to ceftazidime/avibactam.^14,15^ Indeed, meropenem/vaborbactam shows consistent *in vitro* activity against both engineered and clinical KPC-*Kp* regardless of KPC subtype, and has been used to treat patients who have failed treatment with ceftazidime/avibactam.^7,16^ Combining ceftazidime/avibactam with other agents has been hypothesized to improve outcomes and suppress treatment-emergent resistance; however, the available clinical data are conflicting.^8,17–20^ In fact, the inherent biases of the existing retrospective clinical studies have made determining the impact of combination therapy difficult. We hypothesize that the heterogeneity of combination regimens employed clinically may further mask potential benefits, and that combinations intentionally selected by underlying mechanisms of resistance can improve *in vitro* killing.

One important mechanism that impacts BL/BLI *in vitro* activity is mutations in *ompK36*, a gene that encodes the outer membrane porin OmpK36. This porin is responsible for entry of nutrients and other molecules, including vaborbactam, into the periplasmic space.^14,21^*ompK36* genotypes among KPC-*Kp* are heterogeneous and vary within specific geographical regions. A recent global survey identified an insertion or other mutations in the L3 loop of *ompK36* in 24% of *K. pneumoniae* genomes, and a glycine-aspartic acid duplication (GD) as the most common mutation.^22^ This specific mutation results in a 6 bp insertion within the L3 loop that leads to an inner porin channel constriction that reduces the intake of nutrients and antibiotics into the periplasmic space.^21,23^ Another common mutation is IS*5*, a mobile DNA sequence often located in the *ompK36* promoter region, which reduces *ompK36* expression.^24–26^ The prevalence of IS*5* insertions across all KPC-*Kp* is not known; however, isolates from 53% of patients infected with KPC-*Kp* at a single centre harboured IS*5* insertions.^27^ Both IS*5* and GD have been associated with higher meropenem/vaborbactam MICs and reduced bacterial killing against KPC-*Kp* in *in vitro* studies.^10,15,16^ Combination therapy with meropenem/vaborbactam appears to be less common than with ceftazidime/avibactam; however, combination regimens remain common in clinical practice.^8^ Limited reports have described the utility of meropenem/vaborbactam combination therapy; however, whether combinations are impacted by the presence of *ompK36* porin mutations remains unknown.^7,8^

Identification of molecular mechanisms of resistance associated with treatment failure of ceftazidime/avibactam or meropenem/vaborbactam has motivated interest in the use of combination therapy with these agents. To close the gap between clinical practice and current recommendations, we aimed to characterize the *in vitro* activity of ceftazidime/avibactam and meropenem/vaborbactam, alone and in combination with other agents predicted to have *in vitro* activity against KPC-*Kp*. We propose that *ompK36* mutations mediate *in vitro* killing and synergy of these drug combinations. Understanding the interplay between resistance genes and antibiotic synergy may be useful in the selection of combination regimens for treatment of KPC-*Kp* infections.

## Materials and methods

### KPC-Kp isolates

Fifteen clinical isolates—from blood (*n* = 11), respiratory (*n* = 3) or urine (*n* = 1) cultures—collected from patients not previously treated with ceftazidime/avibactam or meropenem/vaborbactam were selected from University of Pittsburgh Medical Center biorepositories. All isolates were stored at −80°C and subcultured twice on Mueller–Hinton agar (MHA; Becton, Dickinson, & Company, Sparks, MD, USA) prior to testing. WGS was performed on an Illumina platform as previously described.^28^ Species, ST and KPC subtype were confirmed with WGS analyses. *ompK36* genotypes were denoted as WT or mutant. Mutant *ompK36* genotypes were categorized as IS*5* and GD duplication as described previously.^15^

### Susceptibility testing

MICs were determined in triplicate by standard methods for broth microdilution in CAMHB (Becton, Dickinson, & Company, Sparks, MD, USA) or agar dilution on Mueller–Hinton agar plates with a starting inoculum of 5 × 10^5^ cfu/mL; consensus MICs were reported as the mode. Susceptibility was defined according to CLSI interpretive criteria.^29^ Tested concentrations for broth microdilution of ceftazidime/avibactam and meropenem/vaborbactam were in the ranges 0.12–256 mg/L and 0.008–8 mg/L, respectively. Avibactam and vaborbactam were tested at fixed concentrations of 4 and 8 mg/L, respectively. Broth microdilution was also performed for gentamicin (0.06–64 mg/L), tigecycline (0.03–32 mg/L), ceftazidime (0.5–512 mg/L) and meropenem (0.06–64 mg/L). Agar dilution was performed for colistin (0.125–128 mg/L) and fosfomycin plus glucose-6-phosphate (G6P) (0.25–256 mg/L plus a fixed concentration of 25 mg/L G6P). Quality control was assessed using *Pseudomonas aeruginosa* ATCC 27853, *K. pneumoniae* ATCC 700603 and *Escherichia coli* ATCC 25922, and results were reported only when MICs for control strains were within acceptable ranges.

### In vitro killing activity

Time-kill assays were performed for each isolate in the presence of ceftazidime/avibactam and meropenem/vaborbactam alone and in combination with various agents. Representative killing-curves were selected from isolates tested in at least duplicate to determine bactericidal and synergistic activity. Prior to testing, each isolate was grown overnight in CAMHB at 37°C with shaking. An initial inoculum of 1 × 10^8^ cfu/mL was selected to represent infections associated with high bacterial burdens that are most difficult to eradicate, which includes ventilator-associated pneumonia.^30,31^ Ceftazidime/avibactam and meropenem/vaborbactam were tested at concentrations of 1 × MIC and 4 × MIC alone. Additional flasks were prepared containing 1 × MIC of either drug in combination with steady-state, clinically relevant concentrations of colistin (2 mg/L), gentamicin (2 mg/L), tigecycline (2 mg/L), meropenem (8 mg/L) and fosfomycin + G6P (100 mg/L + 25 mg/L) consistent with our prior investigations.^18,24^ Each isolate was also tested in a drug-free control flask for validation of microbial growth. All flasks were incubated at 37°C with 200 rpm shaking. Samples were taken at 0, 2, 6, 10 and 24 h, serially diluted, plated on Mueller–Hinton agar plates, and incubated overnight at 37°C. Colonies were enumerated and reported as cfu/mL. Bactericidal activity was defined as ≥3 log-kill at 24 h compared with the starting inoculum. Synergy and antagonism were defined as ≥2 or <2 log-kill at 24 h compared with the most active single agent, respectively.^18^ Regrowth was defined as the presence of bactericidal activity at 10 h that was no longer present at 24 h.^18^

### Statistical analysis

Graphs and statistical analyses were rendered using GraphPad Prism 9 software. Continuous variables were analysed using a Mann–Whitney *U* test. To compare isolates by *ompK36* porin genotype, mean 24 h log-kills were compared for each genotypic group by Student’s *t*-test. A two-tailed *P* value ≤0.05 was considered statistically significant.

## Results

### Characterization of isolates

All KPC-*Kp* were ST258; *ompK36* genotypes were classified as WT, IS*5* or GD (*n* = 5 each). IS*5* disrupted the promoter region in 80% (4/5) of IS*5* isolates. All isolates harboured *ompK35* with a premature stop codon at amino acid 89. KPC subtypes included either KPC-2 (*n* = 11) or KPC-3 (*n* = 4); no other *bla*_KPC_ mutations were identified. Other β-lactamases included SHV-11 (*n* = 5), SHV-12 (*n* = 5), TEM-1 (*n* = 8) and/or OXA-9 (*n* = 8). Six isolates harboured an ESBL (Table 1).

**Table 1.** Characteristics and susceptibility (MICs reported as mg/L) for 15 KPC-*Kp* clinical isolates

*Isolate*	*KPC* s*ubtype*	*ompK36 genotype*	*ompK35 genotype*	*Other β*-*lactamases*	*CAZ MIC*	*CZA MIC*	*MEM MIC*	*MVB MIC*	*C*ST *MIC*	*GEN MIC*	*TGC MIC*	*FOS MIC*
*142*	2	WT	AA89stop	ESBL SHV, TEM	256	0.5	8	0.016	0.5	2	0.5	8
*347*	2		AA89stop	ESBL SHV, TEM	512	1	16	0.016	0.5	1	0.5	8
*972*	2		AA89stop	SHV-12, TEM-1, OXA-9	256	1	64	0.125	0.5	2	2	64
*1439*	3		AA89stop	SHV-11, TEM-1, OXA-9	256	2	16	0.032	0.5	0.5	1	16
*1627*	3		AA89stop	SHV-11, TEM-1, OXA-9	512	2	16	0.016	0.5	0.5	1	8
*20*	2	IS*5*	AA89stop	SHV-11, SHV-12, TEM-1, OXA-9	256	2	>64	0.5	0.25	32	1	32
*685*	2		AA89stop	SHV-12. TEM-1, OXA-9	256	2	>64	4	2	>64	1	128
*873*	2		AA89stop	ESBL SHV, TEM	512	2	64	0.064	0.5	0.5	0.5	16
*913*	3		AA89stop	SHV-12. TEM-1, OXA-9	512	1	32	0.5	0.25	>64	1	128
*1421*	3		AA89stop	SHV-11. TEM-1, OXA-9	256	2	>64	0.25	0.5	1	1	128
*155*	2	GD	AA89stop	ESBL SHV, TEM	>512	2	>64	1	0.5	0.5	0.5	16
*436*	2		AA89stop	ESBL SHV, TEM	512	2	>64	0.25	0.5	0.5	1	32
*669*	2		AA89stop	ESBL SHV, TEM	256	1	>64	0.25	0.5	2	2	64
*705*	2		AA89stop	SHV-12, TEM-1, OXA-9	>512	4	>64	1	0.25	0.5	1	64
*992*	2		AA89stop	SHV-11	64	1	>64	0.25	0.5	0.5	1	32

CAZ, ceftazidime; CST, colistin; CZA, ceftazidime/avibactam; FOS, fosfomycin; GD, glycine-aspartic acid duplication; GEN, gentamicin; KPC, *Klebsiella pneumoniae* carbapenemase; MEM, meropenem; MVB, meropenem/vaborbactam; TGC, tigecycline.

All isolates were susceptible to ceftazidime/avibactam (median = 2, range = 0.5–4 mg/L) and meropenem/vaborbactam (0.25, 0.016–4 mg/L); none were resistant to colistin (0.5, 0.25–2 mg/L). Median (range) MICs for gentamicin were 1 (0.5 to >64 mg/L); three isolates were non-susceptible and each were IS*5* genotypes. Median (range) MICs for tigecycline and fosfomycin were 1 (0.5–2) and 32 (8–128) mg/L, respectively. Median MICs were significantly higher for *ompK36* mutant isolates versus WT tested against meropenem/vaborbactam (0.375 versus 0.016 mg/L; *P* = 0.001), but not ceftazidime/avibactam (1 versus 2 mg/L; *P* = 0.27). No significant differences in median MICs were observed between KPC-2 and KPC-3 isolates tested against either meropenem/vaborbactam or ceftazidime/avibactam (Table 1).

### In vitro time-kill analyses

**Figure 1.**
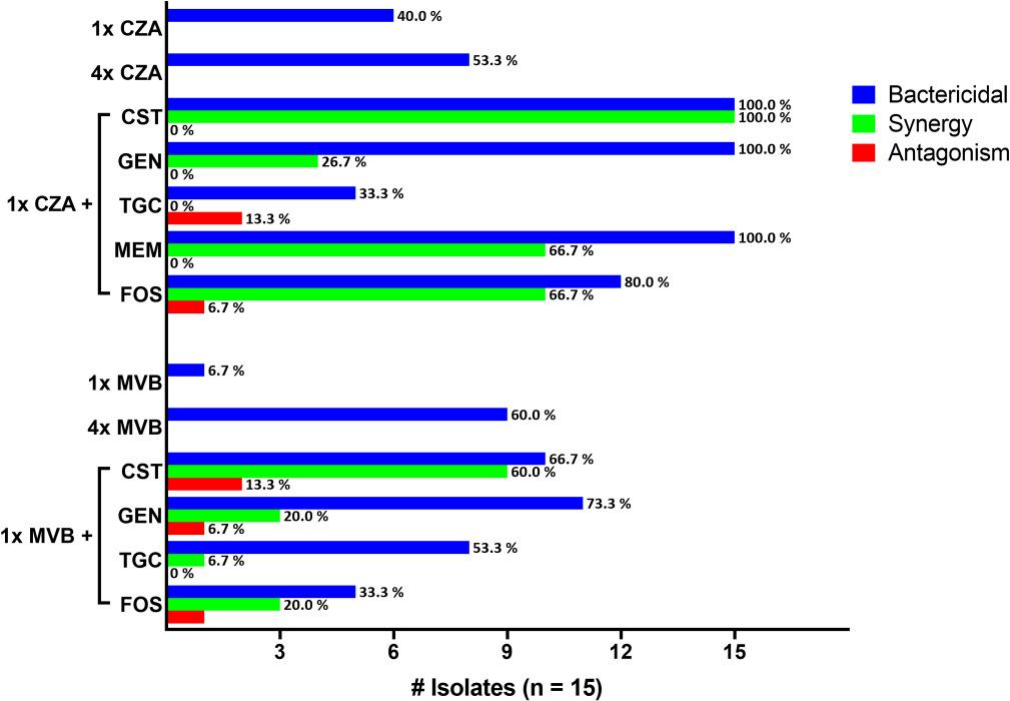
Proportion of bactericidal, synergistic and antagonistic interactions for isolates tested against ceftazidime/avibactam (CZA) or meropenem/vaborbactam (MVB) at 1× and 4×MIC as single agents, and at 1×MIC in combination with colistin (CST), gentamicin (GEN), tigecycline (TGC), meropenem (MEM) or fosfomycin (FOS). Bactericidal activity was defined as ≥3 log-kill from time 0 at 24 h; synergy was defined as ≥2 log-kill in combination compared with most active single agent. Antagonism was defined as at least 2 log less killing in combination compared with the most active single agent.

The mean (SD) starting inoculum achieved in time-kill assays was 7.95 log_10_ (± 0.15) cfu/mL. Ceftazidime/avibactam 1 ×  and 4 ×MIC concentrations were bactericidal against 40% and 53% of isolates, respectively. Corresponding rates for meropenem/vaborbactam at 1 ×  and 4 × MIC were 7% and 60%, respectively. Across other single agents, gentamicin and tigecycline were bactericidal against 53% and 13% of isolates, respectively; colistin, meropenem and fosfomycin were not bactericidal against any isolates (Figure 1). No significant differences in killing activity were observed among single drugs stratified by *ompK36* genotype or KPC subtype.

In combination with ceftazidime/avibactam 1 × MIC, colistin, gentamicin and meropenem were bactericidal against all isolates; mean log-kills were −7.61, −6.28 and −5.67, respectively. Enhanced killing of ceftazidime/avibactam plus colistin or meropenem was attributable to synergy in 100% and 67% of isolates, respectively. By comparison, ceftazidime/avibactam plus gentamicin was only synergistic against 27% of isolates. Ceftazidime/avibactam plus fosfomycin was synergistic or bactericidal in 67% and 80% of isolates, respectively. Combinations with tigecycline did not result in synergistic killing against any isolate, and were antagonistic against 13% (Figure 1).

**Figure 2.**
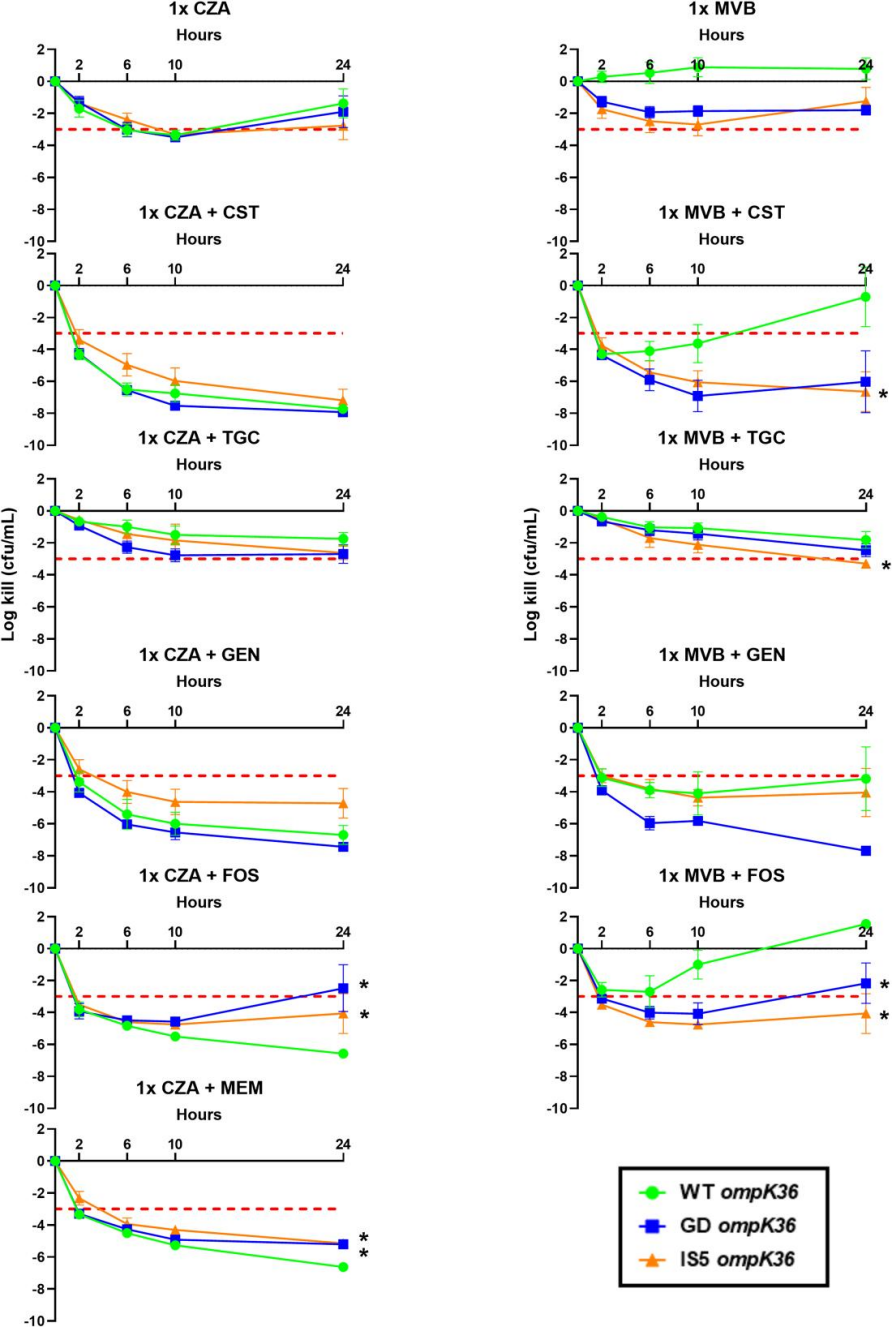
Mean ± SE 24 h time-kill curves for *ompK36* WT (*n* = 5), GD (*n* = 5) and IS*5* (*n* = 5) isolates tested against ceftazidime/avibactam (CZA, left) or meropenem/vaborbactam (MVB, right) at 1×MIC plus colistin (CST), gentamicin (GEN), tigecycline (TGC), fosfomycin (FOS) or meropenem (MEM). Bactericidal activity was defined as ≥3 log-kill from time 0 at 24 h and is represented by the horizontal dotted line. Significant change in log-kill (*P* < 0.05) compared with WT denoted by *.

Overall, the *in vitro* killing with ceftazidime/avibactam plus colistin, gentamicin and tigecycline did not vary with respect to *ompK36* genotype (Figure 2). On the other hand, 24 h log-kills were significantly less for ceftazidime/avibactam plus meropenem or fosfomycin against isolates with *ompK36* mutations. Specifically, for ceftazidime/avibactam plus meropenem combinations, mean log-kills were lower for IS*5* (−5.15) and GD (−5.21) mutants compared with WT (−6.62; *P* < 0.001 and *P* = 0.002, respectively). Similar observations were identified for ceftazidime/avibactam plus fosfomycin against IS*5* and GD mutants (−5.52 and −2.47, respectively) versus WT (−6.58 log; *P* < 0.001 and *P* = 0.024, respectively). Bacterial regrowth was observed against *ompK36* mutants tested against ceftazidime/avibactam plus fosfomycin, but not ceftazidime/avibactam plus meropenem.

In combination with meropenem/vaborbactam 1 × MIC, rates of bactericidal activity ranged from 33% to 73% across combinations (Figure 1). The combinations resulting in the greatest mean log-kills were meropenem/vaborbactam plus colistin (−4.20) and gentamicin (−5.36). Combinations with tigecycline (−2.45) and fosfomycin (−1.21) demonstrated less killing. Rates of synergy were higher for meropenem/vaborbactam plus colistin (60%) than all other combinations combined (16%; *P* = 0.002). *In vitro* killing of the meropenem/vaborbactam plus colistin combination varied significantly against *ompK36* mutant isolates (mean 24 h log-kill = −6.65) compared with WT isolates (−0.70; *P* = 0.03; Figure 2). Bacterial regrowth was identified in 60% (3/5) of WT isolates exposed to meropenem/vaborbactam plus colistin compared with 20% (2/10) of *ompK36* mutant isolates. Killing activity was also greater with meropenem/vaborbactam plus fosfomycin against mutant compared with WT isolates (−3.12 versus 1.55 log; *P* = 0.003); however, bacterial regrowth was common in the presence of both fosfomycin alone and in combination regimens. Porin genotypes did not significantly impact the *in vitro* killing activity of meropenem/vaborbactam plus gentamicin or tigecycline.

## Discussion

The aim of this study was to compare the *in vitro* killing activity of combination regimens containing ceftazidime/avibactam or meropenem/vaborbactam against KPC-*Kp* clinical isolates with varying *ompK36* genotypes. Our results support the hypothesis that *ompK36* plays a significant role in the killing capacity of ceftazidime/avibactam and meropenem/vaborbactam with other agents. Whereas enhanced killing was achieved for meropenem/vaborbactam in combination with colistin and fosfomycin against KPC-*Kp* with porin mutations, killing was attenuated for ceftazidime/avibactam in combination with meropenem and fosfomycin under the same conditions. KPC subtype was not found to be a determinant of bacterial killing for combination regimens, although isolates tested in this study were enriched for *ompK36* mutants, which are more commonly associated with KPC-2 than KPC-3.^15^

Studies of ceftazidime/avibactam combination therapy in the literature have become more common as treatment failures with ceftazidime/avibactam monotherapy have been reported and linked to the emergence of resistance.^6–8,11,12^ A retrospective cohort study of 62 critically ill patients found that those treated with ceftazidime/avibactam plus a carbapenem, fosfomycin or tigecycline (*n* = 41) had significantly lower 30 day mortality than those treated with ceftazidime/avibactam alone (*n* = 21; 24.4% versus 47.6%, *P* = 0.028). Notably, combinations with gentamicin and colistin were not evaluated.^17^ A rabbit osteomyelitis model demonstrated favourable results using combinations of ceftazidime/avibactam plus colistin and gentamicin, among other agents, resulting in rapid and complete kill in time-kill analyses and achievement of bone sterilization *in vivo.*^32,33^ Our data demonstrate that ceftazidime/avibactam alone at 1 ×  and 4 ×  the MIC exhibited only modest killing activity against KPC-*Kp* (−2.01 and −2.51 mean 24 h log-kills, respectively), whereas combinations with colistin, meropenem and fosfomycin resulted in synergistic and bactericidal killing, with mean log kills of −7.61, −5.67 and −4.86, respectively. Ceftazidime/avibactam plus gentamicin was also bactericidal, which was at least in part attributable to the rapid bactericidal activity of gentamicin alone.

Importantly, the bactericidal effects of ceftazidime/avibactam plus colistin were not affected by *ompK36* mutations; however, combinations with meropenem and fosfomycin were. This is likely due to the fact that both meropenem and fosfomycin gain entry to the periplasmic space through outer membrane porins. Porin loss and loss-of-function mutations have been associated with reduced susceptibility and treatment failure with meropenem, both with and without vaborbactam.^15,26,34,35^ Despite the differential killing noted for ceftazidime/avibactam plus meropenem across KPC-*Kp* porin genotypes, it is important to note that the mean log-kill against porin mutants was −5.18, well below the bactericidal threshold. The combination of ceftazidime/avibactam plus a carbapenem capitalizes on collateral sensitivity to meropenem in the presence of KPC variants, and can potentially suppress treatment-emergent resistance.^20,36^ The combination likely imparts safety advantages over combinations with colistin or gentamicin, which are commonly associated with nephrotoxicity and vestibulotoxicity.^37,38^ Treatment successes with ceftazidime/avibactam plus a carbapenem have been reported in cases of critically ill patients, further supporting a role clinically.^17,19^ The relationship between fosfomycin and porin genotypes is less well understood, but recent studies suggest that fosfomycin permeates well through *E. coli* OmpF, a homologue of *K. pneumoniae* OmpK35.^39^ Ceftazidime/avibactam in combination with fosfomycin has been used successfully in patients with KPC-*Kp* bloodstream infections and is associated with reduced recurrence and secondary infection rates compared with treatment with ceftazidime/avibactam alone.^40^

Meropenem/vaborbactam combination therapy has been utilized in the setting of salvage therapy when ceftazidime/avibactam resistance arises.^7,8^ As with ceftazidime/avibactam, meropenem/vaborbactam alone demonstrated only modest killing activity at concentrations of 1 × MIC (mean log-kill −0.84) and 4 × MIC (−2.87); however, these concentrations are well below plasma trough levels achieved in patients receiving treatment.^41^ Combinations with colistin and gentamicin were highly effective as evidenced by mean log-kills of −4.20 and −5.36, respectively. Colistin exhibited synergy against 60% of isolates, whereas gentamicin with meropenem/vaborbactam was not synergistic These findings relate to a key outcome of our study in that the killing activity of meropenem/vaborbactam was potentiated in combination with colistin and fosfomycin against KPC-*Kp* with porin mutations. It is well known that porin mutations limit the entry of meropenem/vaborbactam into the periplasmic space; the addition of a second agent, especially a membrane-depolarizing drug like colistin, may create additional entry points for meropenem/vaborbactam to reach its site of action. Another potential mechanism for enhanced killing is the fitness cost associated with porin mutations in KPC-*Kp*, which may be exacerbated by drug pressure in the presence of two or more agents.^21^ These data support a potential role for meropenem/vaborbactam combination therapy for treatment of isolates known, or suspected to, harbour *ompK36* porin mutations. As we reported previously, median meropenem/vaborbactam MICs are higher against KPC-*Kp* with *ompK36* GD (0.5 mg/L) and IS*5* (0.5 mg/L) versus WT (0.03 mg/L; *P* = <0.001) genotypes.^15^ In the absence of real-time molecular characterization, it is plausible that meropenem/vaborbactam MICs may be used as a surrogate marker for porin genotype, and accordingly trigger consideration of combination regimens with colistin or fosfomycin in select cases.

Importantly, the addition of tigecycline does not routinely result in synergy with either ceftazidime/avibactam or meropenem/vaborbactam. In fact, tigecycline combinations were antagonistic against two isolates, and mean log-kills failed to reach bactericidal thresholds. Contradictory accounts of synergy with tigecycline have been published, with some studies demonstrating recovered sensitivity to β-lactams in combination with tigecycline, whereas other studies have demonstrated antagonism and treatment failures.^20,42,43^ Our data show that both ceftazidime/avibactam and meropenem/vaborbactam in combination with colistin or gentamicin were more potent *in vitro* than combinations containing tigecycline. Although many factors influence the decision to select a combination regimen in clinical practice, it is notable that tigecycline-based regimens are often employed.^8,44,45^

Taken together, our results suggest that combination therapy featuring ceftazidime/avibactam or meropenem/vaborbactam may be potentially useful in treating KPC-*Kp* infections; however, not all combinations demonstrate equal activity across tested clinical isolates. Ceftazidime/avibactam in combination with colistin or meropenem demonstrates potent *in vitro* bactericidal and synergistic killing even in the presence of porin mutations. Conversely, meropenem/vaborbactam killing was potentiated by colistin and fosfomycin in the presence, but not the absence of porin mutations. Although these results are meaningful in select cases, it is important to acknowledge that use of potentially toxic agents like colistin or gentamicin in combination may negate the safety advantages imparted by prioritizing the BL/BLI agents in clinical practice.^37,38^ Thus, our results should be viewed in light of contemporary clinical data showing that both ceftazidime/avibactam and meropenem/vaborbactam are superior treatment options when compared with polymyxin-based combinations.^46,47^ In the only comparative study to date, ceftazidime/avibactam was used in combination more commonly than meropenem/vaborbactam resulting in higher rates of adverse events.^8^ It is also important to acknowledge that *ompK36* porin status is not typically known at the time of treatment initiation; however, we have previously shown that meropenem/vaborbactam MICs  ≥ 0.5 mg/L among KPC-*Kp* are highly predictive of porin mutations.^14,15^ Next, we tested ceftazidime/avibactam and meropenem/vaborbactam in combination at concentrations equivalent to the MIC for each isolate to detect synergy, but concentrations achieved *in vivo* are often much higher. We also note that mean log-kills of ceftazidime/avibactam at 4 ×MIC were lower in the current study than our previous investigation,^15^ likely due to the higher inoculum employed here. The inoculum used in the current study is consistent with high-burden infections like ventilator associated pneumonia where combination therapy may be considered to suppress bacterial burdens below the frequency of mutation selection.^48^ Taking these factors together, our findings require further validation before clinical implementation. Future experiments that test dynamic, humanized antibiotic exposures in preclinical *in vitro* and *in vivo* models of combination therapy will help close these knowledge gaps.
